# A fusion approach of YOLOv8 and CNN-Transformer for End-to-End road anomaly detection

**DOI:** 10.1038/s41598-025-29718-4

**Published:** 2025-11-25

**Authors:** Sarfaraz Abdul Sattar Natha, Mohammad Siraj, Saif A. Alsaif, Fahad Farooq, Admali Shah, Maqsood Mahmud

**Affiliations:** 1 Department of Software Engineering and Electronics, Sir Syed University of Engineering & Technology, Karachi, Pakistan; 2 Department of Electrical Engineering, College of Engineering, King Saud University, Riyadh, 11543 Saudi Arabia; 3 Department of Electrical Engineering, School of Computing, 2-24 York Street , Northern Ireland BT15 1AP Belfast, UK

**Keywords:** Smart transportation system, CNN, Deep learning, Transfer learning, YOLO, Road anomaly detection, Engineering, Mathematics and computing

## Abstract

Surveillance cameras are common in both the private and public sectors for security and monitoring, and closed-circuit television (CCTV) systems are used for surveillance, generating large amounts of video data that cannot be manually monitored 24/7. The traditional approach to analysis is time-consuming and inefficient, and there is a growing need for automated surveillance systems that can recognize and classify anomalies. The research area that has been the most challenging to solve is AD systems that detect anomalies in data that is not structured according to the normal patterns. RNNs are slow and have difficulty identifying anomalies in the road that occur in multiple frames at the same time, whereas CNNs are limited in extracting temporal features from objects and generally disregard the background noise in video frames. In this study, a new framework for background removal is presented that removes the irrelevant background elements during object recognition. This framework saves temporal and spatial information over frames and uses YOLOv8 and a spatial-temporal adaptive fusion method with an end-to-end model based on a CNN encoder and a Transformer decoder for parallel video investigation. The proposed method was tested on the UCF Crime dataset and a custom Road Anomaly Dataset (RAD), and the accuracy of the framework was 89.90% on the UCF Crime dataset and 98.28% on the RAD dataset.

## Introduction

Security has become an important concern in several areas where crime rates are rising. Surveillance devices not only create a significant amount of surveillance images, but they also do not provide people with a complete security guarantee^1^. The development of CCTV surveillance has been used globally to maintain public safety, help law enforcement, increase personal security, solve crimes, identify traffic violations, and manage traffic^2^ with advances in networks, storage, and processing systems. The massive amounts of real-time video data, manual analysis by humans is impractical and time-consuming; therefore, we needed automated technologies to quickly and effectively analyze video data are required for CCTV systems to be effective. These abnormal events include road accidents, explosions, theft, physical fights, and robbery^3^.These are the most difficult to identify in surveillance videos. Anomaly detection (AD) systems have become one of the most challenging areas of research into identifying data that deviates from standard patterns^4^. Various sources can be used to analyze and interpret the context of abnormal events^5,6^. Anomaly detection (AD) identifies anomalies in data^7^. In smart cities, AD systems enhance security by detecting abnormal activity and associating the event with the location. AD has a wide range of applications in fraud detection^8^, fault diagnosis^9^, and human activity recognition^10^, network sensor monitoring^11^, and medical imaging^12^.However the application of AD in real-world environments is still challenging^13^, particularly in smart cities, as dynamic and complex conditions exacerbate the challenge^14^. AD systems are important for situational awareness, but the lack of annotated data continues to limit accurate anomaly detection^15,16^. Another important challenge is data complexity: The factors contributing to anomalous events make it difficult to detect abnormal patterns in an intelligent environment^17,18^. The imbalanced nature of AD problems also poses a unique assessment challenge, and the selection of the appropriate evaluation metrics to address the imbalance is crucial^19^. The aim of this study is to accurately localize anomalous events in both space and time in a sequence of frames. Determining a global boundary or model that captures all the normal behaviors is difficult^20^. The most challenging anomalies to identify are intelligent anomalies, or adversarial samples, which are designed to appear like normal patterns. Here, we present an advanced framework that integrates deep learning with background subtraction to detect anomalies on roads. Background subtraction produces bounding box masks, which successfully isolate moving objects from the scene, and a CNN encoder captures the spatial information (e.g., object size and location) to model how these objects function.

- The suggested framework uses YOLOv8s to automatically eliminate road backgrounds, reducing interference and increasing the precision of object detection.
- This fusion approach uses a CNN encoder and a Transformer decoder to extract spatiotemporal dependencies between objects to precisely detect road anomalies, as opposed to earlier methods that aggregate or concatenate spatiotemporal features. This technique takes video sequences at various time intervals and extracts discriminative information.
- This framework also allows for the parallel processing of consecutive video frames to scale large and complex datasets and enable real-time detection across multiple frames.
- The experimental evaluation demonstrates that this framework is reliable and performs better than earlier approaches, achieving 98.28% accuracy on the RAD dataset and 89.90% accuracy on the UCF Crime dataset.

This paper is organized as follows: Sect. 2 provides a summary of current developments in road anomaly detection. Section 4 presents the experimental results obtained from the model, while Sect. 3 describes the suggested framework. Section 5 brings the study to a close and outlines potential directions for future research.

## Related work

In this session, an overview of key concepts and techniques in the latest methods for anomaly detection, specifically applied to road anomalies, will be provided.

### Machine learning approach

Traditional approach methods for anomaly detection typically involve machine learning approaches. The SVM and K-Nearest Neighbor (KNN) algorithms are categorized for driver behavior at traffic signals, specifically examining safe versus unsafe stopping responses at the yellow light in dilemma zones throughout rural, suburban, and urban areas^21^. KNN and Linear Discriminant Analysis achieve accuracies of 90.1% and 89.4% respectively. However, the cubic kernel underperforms, and the Gaussian kernel’s high computational demand may hinder real-time use. The model uses a Random Forest classifier to predict accidents by differentiating between accidents and non-accidents. In this study^22^. The authors proposed a model that makes use of historical weather and accident data gathered from the road network. In addition to a remarkable specificity of 0.97 and a low false positive rate of just 3%, the findings of this technique show an accuracy rate of 73%. However, the model’s very low sensitivity of 0.08 suggests that it would miss a sizable portion of possible accidents.

K-Means clustering is applied to cluster the road accident variables based on the similarity^23^, which improves the dataset by generating another feature for training. The features are then classified into severity levels using Random Forest, which performs better than traditional models (SVM, KNN, and Logistic Regression). Elakiya et al.^24^ suggested a method for detecting behavioral patterns based on the combination of KNN with median filtering. Similarly, Chriki et al.^25^ presented a framework that combines deep features from GoogleNet with traditional hand-crafted descriptors, such as HOG and HOG3D, and is executed using a structured process from dataset collection to UAV surveillance.

### Deep learning approach

Traditional methods are combined with new deep learning-based methods. In this study, Shoaib et al.^26^, proposed a technique that improves detection of important changes in motion regions in video frames using background removal and an attention mechanism, which is able to classify events as normal or abnormal on the UCF Crime public dataset with 96.89% accuracy using a 3D CNN. While this strategy works, it comes with several problems. The approach is sensitive to the quality of the input data, as is the case in real-world applications. In an effort to develop more effective and robust surveillance systems, two new models were recently suggested to optimize detection performance and maintain flexibility across different scenarios^27^. R. Nawaratne et al. also recommended a deep learning-based technique for real-time video surveillance anomaly identification and localization^28^. Although the approach is unique, its middling detection accuracy of 85% suggests that performance might be enhanced. A probability model efficiently extracts the size, velocity, and position features of video frames by assigning weights to expected particles based on how likely they are to be associated with anomalies^29^. The framework achieves lower processing time and Equal Error Rate (EER) than current state-of-the-art algorithms in testing on the UCSD and LIVE datasets. The authors of the study^30^ deployed CNNs that were first trained for object classification to a variety of tasks, including scene classification, visual instance retrieval, and attribute detection. A 2D CNN that had previously been trained on image classification datasets was modified in another work^31^. to extract features from various regions of input images. In a similar manner, in this study, U. Arul et al.^32^ present an adaptive recurrent neural network designed for detecting anomalies in video images. This innovative approach merges a recurrent neural network with a crystal structure algorithm. This method is removed after development, which makes it easier to identify the frames. Long Short-Term Memory (LSTM) networks are frequently used for identifying departures from typical designs in various fields due to their ability to process time-series data with resilience^33^. F. Ding et al.^34^ define the role of LSTMs are also effective for detecting dynamic anomalies due to their ability to model temporal and contextual dependencies. LSTM networks are suitable for trajectory-based anomaly detection, as they have been successful in modeling and predicting object trajectories. Studies using data on container movement have demonstrated the ability of LSTM autoencoders to detect navigational irregularities, which are crucial to maritime safety. Another approach is to use LSTMs to predict normal trajectory patterns where deviations constitute anomalies. However, complex backgrounds with many objects or people often pose challenges for LSTM-based approaches. Motion-related anomalies were detected using a one-class SVM (OCSVM). Connected component analysis was employed to reduce false positives from unexpected motion in low-motion regions, and AUC scores of 76.08 (pixel level) and 97.53 (frame level) were achieved. Furthermore, the authors^35^ proposed the method that compactness and feature representation are critical in image classification and feature learning, as they improve efficiency and streamline the learning process. To address this, S. Lei et al.^36^ introduced a multi-scale feature extraction framework that captures video data at various resolutions, added a Spatial Pyramid Convolution (SPC) module to enhance object recognition at different scales, and added a Weakly Supervised Data Augmentation Network (WSDAN) to improve input images through guided augmentation, which were then processed by a U-Net model, reaching AUC scores of 86.2% and 97.9% on the tested datasets. Nevertheless, it remains an expensive process with many influencing factors and is limited by the perception of the images. YOLO (You Only Look Once) is a popular real-time object detection method because it classifies and localizes in a single forward pass.

The use of ts for anomaly detection in traffic surveillance as well as in security and surveillance. Building on this, to avoid escalation and mitigating possible losses, the Ganagavalli group^37^ implemented a system for video surveillance tracking of automated real-time crime detection and developed optimized algorithms for YOLO frameworks. This design enables the system to detect unlawful activities in real-time, alerting both the public and the authorities. Reporting AUC results of 0.8299 on 14 crime categories and 0.91 for vandalism detection, the system outperformed existing frameworks in precision, F1 score, training loss, and testing loss. Feature extraction sometimes produces false positive results due to capturing poorly relevant information. Using self-attention methods, the Vision Transformer (ViT) is able to detect objects by deciphering global relations within an image. For example, Transformer-based Tailing D was suggested by^38^. TSViT-B/512 variant surpassed the best-performing basic CNN (which obtained 63.54% accuracy) by increasing recall from 91.92% with ResNet-101 to 98.05% with 76.56% accuracy. However, the model still presents shortcomings which, in part, due to the small sample size, may lead to problems with generalization in practical settings. For example, X. Yan et al.^39^ Focused on separating foreground and background in images, and built a model with two encoders, a motion encoder capturing the differences in a sequence of images as motion input, and another encoder that receives the last frame of a static image. These issues highlight the need for further model refinements which aim to improve the accuracy and maintain a level of consistency in the performance across a diverse range of video data of varying temporal lengths. The Strengths and Limitations of previous work are summarized in Table 1.

**Table 1:** Summary of prior studies’ strengths and limitations.

Author, Year, Reference	Strength	Limitation
E. Mujkic et al. 2022^40^	This proposed study makes use of convolutional autoencoders to detect objects that deviate from common patterns. An autoencoder network can be trained to reconstruct typical patterns in agricultural fields with high reconstruction errors in order to identify unknown objects. The PR-AUC was 0.93 using this method.	Autoencoders were used in this model, and although they worked well, they were often lossy and required a lot of processing power. Inaccurate object detection was occasionally caused by the flawed decoding process.
J. W Lee et al. 2024^41^	Achieves strong accuracy across different datasets, is adaptable for various settings, offers flexible model complexity, and provides a valuable benchmark for future studies.	Resource-intensive for complex models, varying results on some datasets, limited testing under real-world conditions, and a lack of emphasis on real-time speed.
K. Razaee et al. 2024^42^	In real-time surveillance, the technique effectively eliminates false positives and negatives with a high accuracy of 94.13% and excellent sensitivity and specificity. Robust anomaly detection is ensured by combining hand-crafted features with deep learning.	The high computational resource requirements of this approach, such as high-performance GPUs, may limit scalability in resource-constrained environments. Large, labeled samples are necessary for training, and dynamic or obstructed conditions may affect tracking effectiveness.

## Methodology

**Fig. 1:**
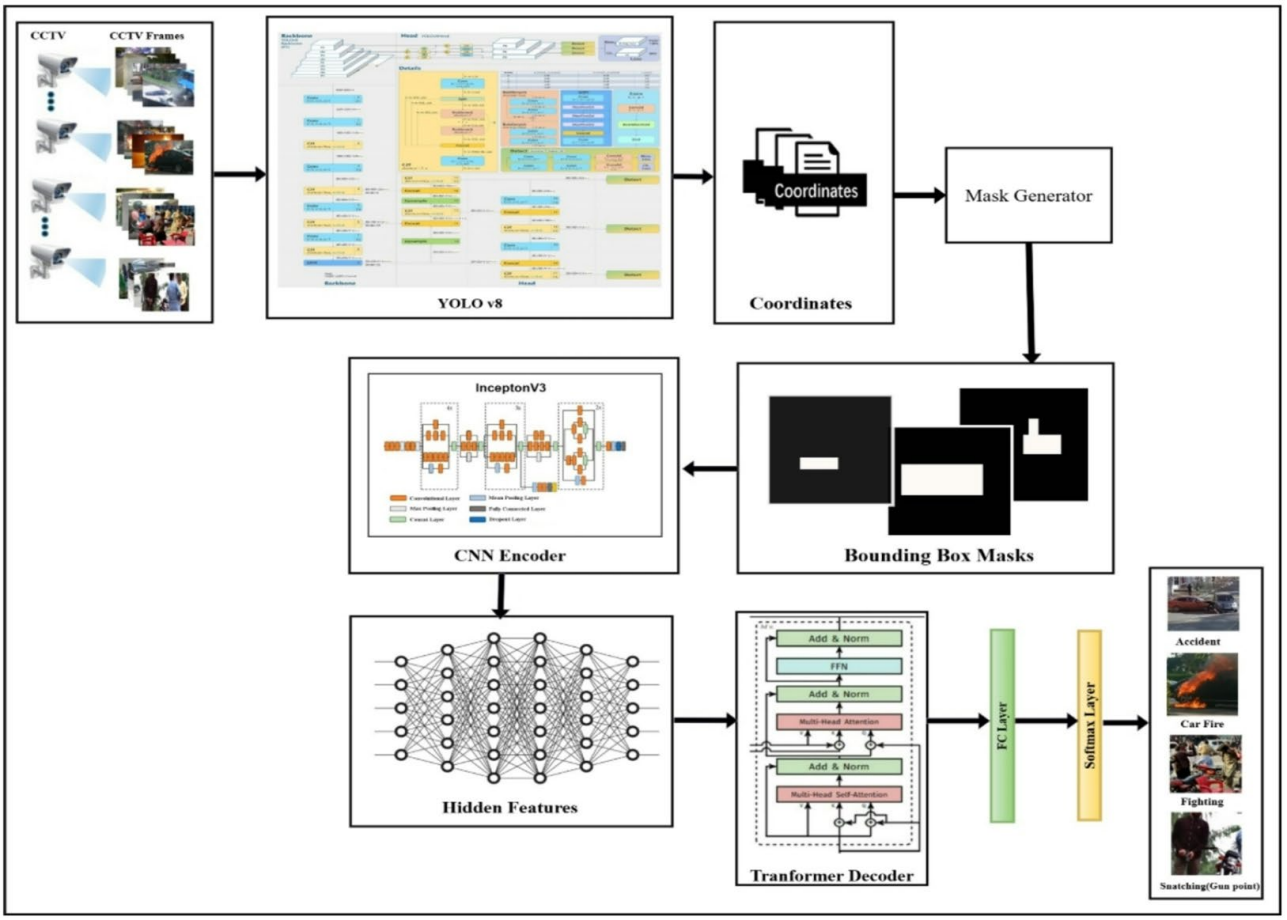
The proposed architecture performs road anomaly detection through a bounding-box-masks extractor.

The proposed frame uses YOLOv8s to detect objects and draw their bounding boxes after the video pre-processing isolates frames from the video frames and the Mask Generator creates the corresponding masks for those bounding boxes, which were separated from the background by the image preprocessor. This background subtraction method reduces the effect of excessive noise, which improves detection accuracy, but traditional detection methods fail due to background interference^43^. The proposed approach overcomes this limitation by using a CNN encoder to capture spatial features from individual frames, such as object size and location, and a Transformer decoder to model temporal dependencies across sequences of frames, fused into a single end-to-end framework., is presented in Fig. 1.

### Bounding box masks extractor

**Fig. 2:**
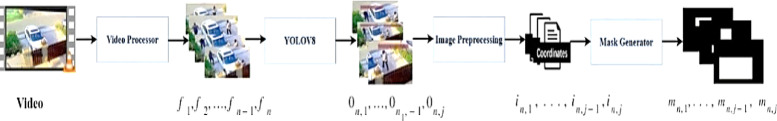
The structure of the Bounding-Box-Masks Generator.

The YOLOv8 object detection method allows real-time processing of video frames and can be used to navigate complex, crowded environments^44^. This model works well for object detection. The Bounding-Box-Masks Generator is an automatic extraction of bounding box masks from the detected substances with the YOLOv8 technique^45^, and Fig. 2 shows its architecture. Videos are processed to obtain frames. Videos are resized to 800 × 600 pixels at 30 frames per second.

The part of the video that contains the detected Road Anomaly is cut to form a 40-frame clip, which lasts for 2 s and retains the original resolution. Each frame in this clip is manually labeled to indicate the presence of a Road Anomaly. The normalized frames, labeled as f1, f2,., fn, are fed into the YOLOv8s model within the Bounding Box Masks Generator to detect all moving objects in images^46^. YOLOv8 utilizes bounding boxes of different sizes to identify substances of various scales and assigns a unique identity to each detected object. All perceived bounding boxes in a separate frame are labeled as o_n,1_, …., o_n, j−1_, o_j_, ensuring that the respective object in the input frames is represented by a single bounding box, with j indicating the j^th^ bounding box. The organization of these bounding boxes is denoted as i_n,1_, …., i_n, j−1_, i_j_ are used to track the detected objects. A Mask Generator creates bounding box masks labeled m_n,1_, …., m_n, j−1_, m_j_. The image preprocessor outputs a tuple of five values(x_n, j_, y_n, j_, w_n, j_, h_n, j_, c_n, j_) representing one bounding box for every object. In this tuple, (x_n, j_) and (y_n, j_) are the coordinates of the center of the bounding box, while w_n, j_ and h_n, j_ indicate its width and height, respectively. The values for x_n, j_ and w_n, j_ range from 0 to 800 pixels, while y_n, j_ and h_n, j_ range from 0 to 600 pixels. The confidence score c_n, j_ indicates the likelihood of an object being present within the bounding box, with scores ranging from 0 to 1. To enable background subtraction, elements are classified into two categories: background and objects. Static components like Highways, buildings, and trees make up the background, whilst moving items like cars, people, and animals make up the objects. Using manually defined bounding boxes. The image preprocessing phase establishes object coordinates. Any bounding box that has a YOLOv8-calculated confidence score c_n, j_ less than 0.55 is initially ignored. The bounding box from neighboring frames is utilized as a stand-in if an item is recognized without one. Only the center coordinates x_n, j_, y_n, j_ and dimensions w_n, j_, h_n, j_ (width and height) of each bounding box are retained, while the confidence score is discarded.

**Fig. 3:**
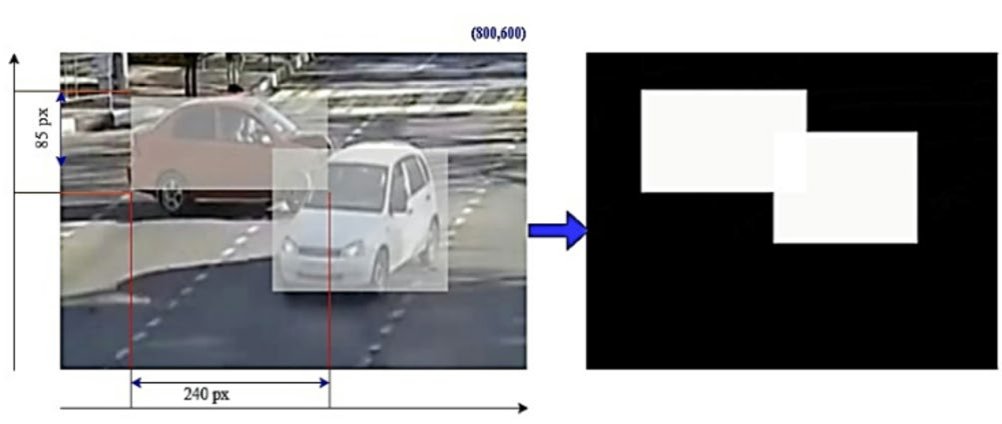
The sample images of the bounding box mask after background subtraction.

**Fig. 4:**
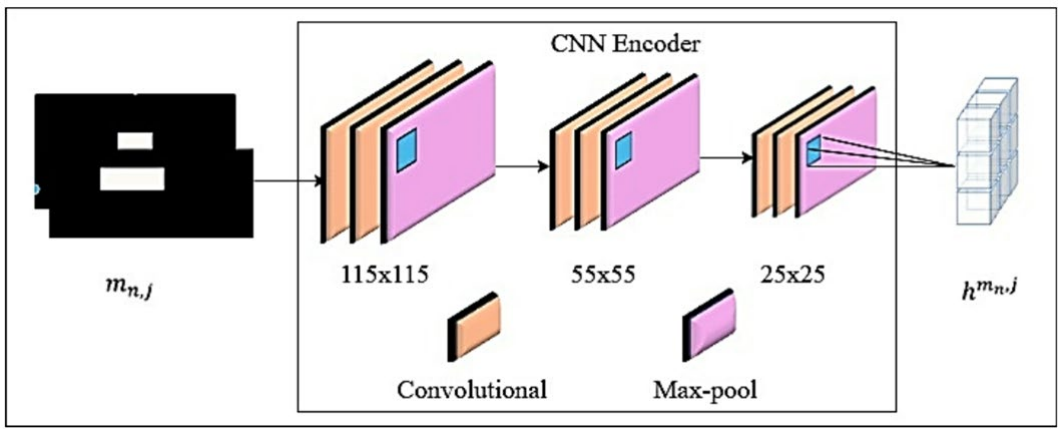
The CNN encoder architecture.

Figure 3 provides an instance of a bounding box mask for an 800 × 600 video frame, where a detected object occupies a region of 85 × 240 pixels (length x width)^47^. Within this area, object pixels are marked in white, and the outstanding pixels are blacked out. After background subtraction, each detected object is signified by a rectangular mask. These saved bounding box m_n,1_, …, m_n, j−1_,m_j_ are then fed into the road anomaly detection system. The YOLOv8 model demonstrates high efficiency in locating road anomalies in video frames, which supports a robust detection step necessary for successful background subtraction. Also, the multi-head attention mechanism in the transformer decoder improves presentation, especially in cases involving multiple object interactions (Fig. 4). This flexibility demonstrates the framework’s capability to detect road anomalies under various lighting conditions. The diversity of the dataset provided a comprehensive and challenging range of scenarios for the evaluation of the road anomaly detection^48^. The framework was able to detect road anomalies in frames from a variety of different environments and conditions, indicating robustness of the framework.

### Road anomaly detector

The proposed model with a CNN encoder is composed of a series of convolutional and max-pooling layers that can process the 224 × 224 pixel input bounding box masks m_n, j_,. The convolutional layers employ 5 × 5 filters with 1 pixel padding to preserve the spatial resolution of the feature maps, and then max-pooling layers with a 3 × 3 filter and a stride of 2 to reduce spatial dimensions and highlight important features. In this architecture, each pair of convolutional layers is followed by a max-pooling layer that decreases the feature map dimensions while preserving critical information. This configuration surpasses the typical CNN structure by stacking several convolutional layers before each pooling operation, improving its capacity to collect spatial data^49^. From each bounding box mask. The CNN encoder successfully extracts spatial features such as object location and size. The feature map sizes are gradually decreased by the encoder, from 115 × 115 to 55 × 55 and finally to 25 × 25. Once spatial features are extracted from all bounding box masks and then sent to the transformer decoder, which analyzes temporal relationships across the bounding box masks to complete anomaly detection^50^. This iterative process of convolution and max pooling in the CNN encoder is expressed in Eq. 1.

1 $$\:{h^m}n,j = M\left( {F\left( {{w_k}\: \times \:\left( {M\left( {F\:({w_k}\: \times \:(\:M(\:F\:\left( {{w_k}\: \times \:\:{m_{n,j}} + {b_k}} \right) + \:{b_k}} \right)} \right) + \:{b_k}))} \right)} \right)$$

**Fig. 5:**
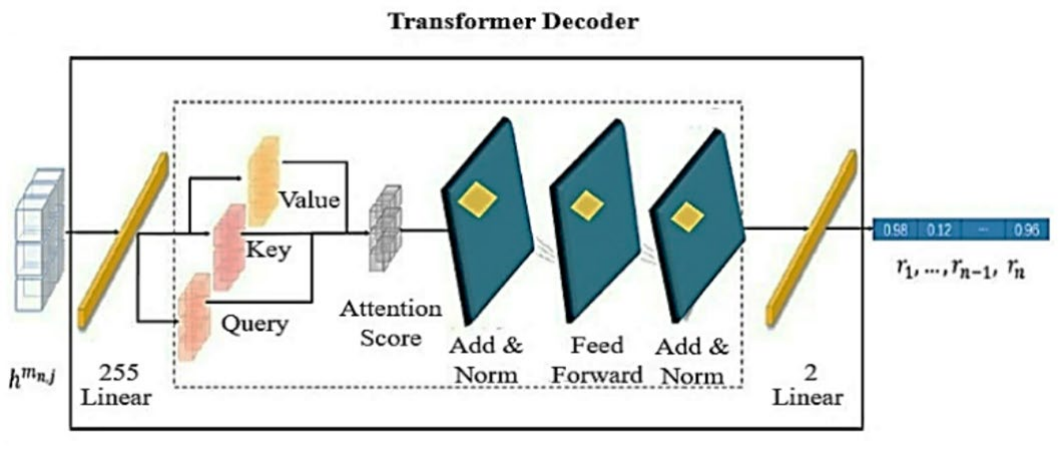
The Transformer Decoder architecture.

Here, F represents the activation function, specifically the Rectified Linear Unit (ReLU), and M represents the max-pooling operation, convolutional weight matrix, and b bias vector. The process begins by applying the kth convolution (w_k_ × m_n, j_ + b_k_) to the input m_n, j_. The result is first passed through the activation function F, and then max-pooling M is applied. This procedure continues across all layers until the last convolution and pooling operations are completed. As shown in Fig. 5, the transformer decoder is built using the output from the CNN encoder as its input. The unseen features presented as h^m^_n, j_ of *m*_n, j_ are generated. This decoder comprises a linear layer, a multi-head attention mechanism, and a feed-forward network. This study transformer decoder includes modifications that distinguish it from the initial Transformer model. The original Transformer architecture consists of both an encoder and a decoder. Additionally, two extra linear layers are incorporated within the decoder^51^. The first linear layer projects the hidden input features into a higher-dimensional space, adjusting them to 255 units for processing in the next layers. The final linear layer, made up of two neurons, serves as the output stage. It transforms the hidden features into a probability distribution across the target classes, providing classification results for multiple video frames of traffic incidents denoted as *r*_1_, …, *r*_*n* − 1_, *r*_n_. The output of each frame is passed through a softmax layer, which limits the values to between 0 and 1, and a value close to 1 indicates a high probability of an anomaly in that frame, and a value close to 0 indicates a low probability. In this study, the multi-head attention mechanism performs parallel processing to derive temporal characteristics from hidden input representations, allowing the model to attend to information at various positions to capture a wide range of temporal dependencies. As stated in Eq. 2, a multi-head attention mechanism is used with eight heads, each with a hidden dimension of 32, in the transformer decoder.

2 $$\:{h}^{m}m,j=Sofmax\left(\frac{\left({h}_{q}^{m}{n}_{,}j\times\:{h}^{m}n,j\right)\times\:\left({h}_{k}^{m}{n}_{,}j\times\:{h}^{m}n,j\right)T}{\sqrt{{d}_{{h}_{k}}^{{m}_{n,j}}}}\right)\times\:\left({h}_{v}^{m}{n}_{,}j\times\:{h}^{m}{n}_{,}j\right)$$

Where h_q_^m^_n, j_, h_k_^m^_n, j_, and h_v_^m^_n, j_ denote the query, key, and value matrices. They are embedded in the unseen input features. The softmax function (.) is practical to the scaled dot result between the query h_q_^m^_n, j_ and key metrics h_k_^m^_n, j_, and it is then multiplied by the value matrix h_v_^m^_n, j_. In addition, d_hk_^m^_n, j,_ and the square root are used to scale the dot product. Here, T is the transpose symbol. To enhance learning stability by using a normalization function and a residual connection following the multi-head attention mechanism and feed-forward network. To modify the hidden features influenced by the attention weights. The feed-forward network includes two linear transformations, with a ReLU activation function.

### InceptionV3

**Fig. 6:**
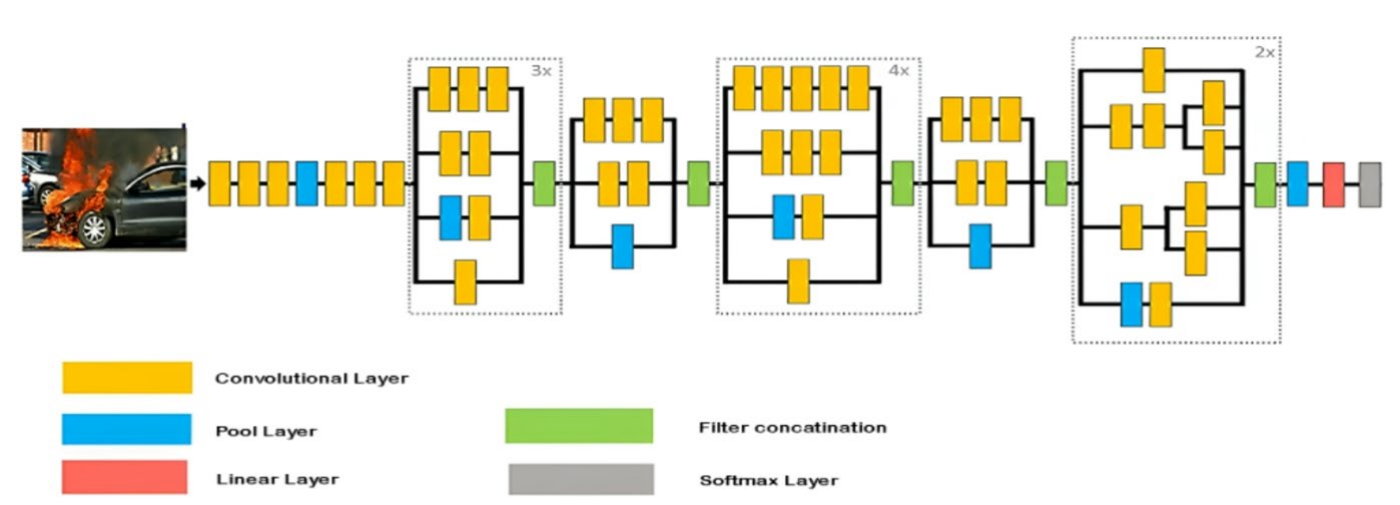
Basic architecture of Inceptionv3.

The Inception model is a type of CNN designed to improve deep neural networks by expanding their width and depth while efficiently utilizing computing resources. It was achieved by approximating a sparser network structure using dense matrix operations, which are well-suited to contemporary technology. The primary aim was to utilize readily available dense components as an approximation of an ideal local sparse design. In addition, the design applies projection and dimensionality reduction techniques to keep computational costs under control when they become excessive^52^. The architecture includes multiple Inception modules, each combining 1 × 1, 3 × 3, and 5 × 5 convolutional operations. The outputs from these different filters are joined together into a single feature vector, which is then passed as input to the next layer. These modules are arranged in sequence, with max-pooling layers (stride of 2) occasionally inserted between them. The structure implemented in this study is shown in Fig. 6.

## Experimental assessment and performance

This section explains the experimental setup and datasets that serve as the basis for our investigation. A comparison is then carried out between our suggested model and several cutting-edge anomaly detection techniques. Furthermore, we provide the outcomes of a methodical assessment of numerous network elements using ablation investigations.

### RAD dataset

**Fig. 7:**
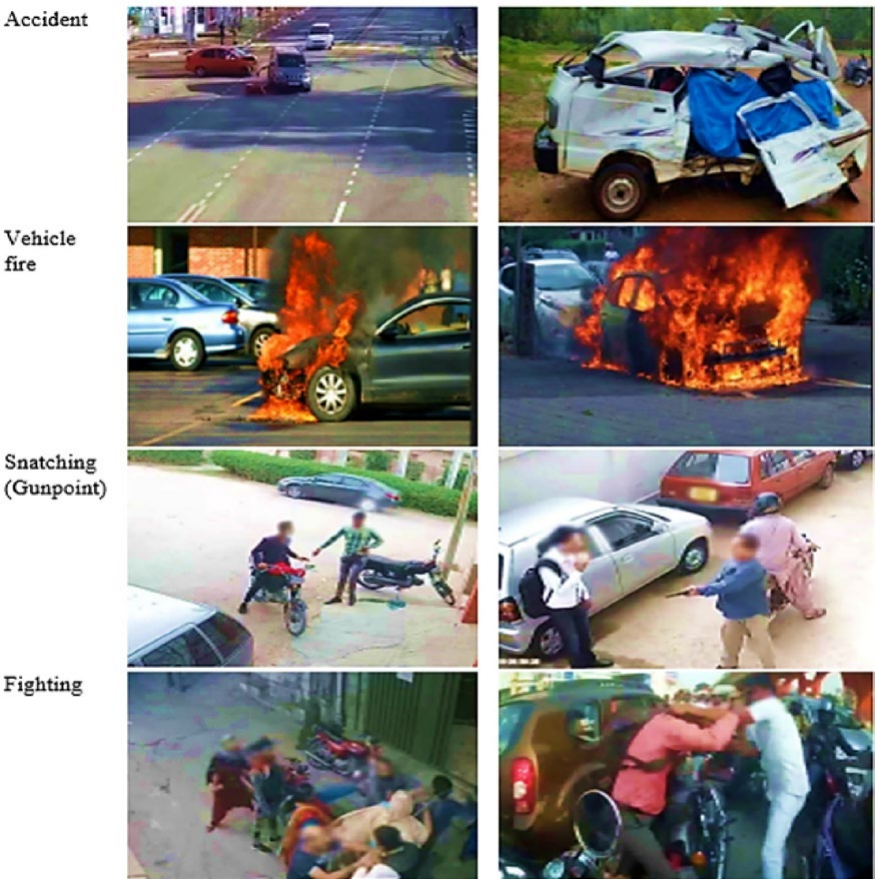
Sample images of the RAD dataset.

The RAD dataset includes various videos and images collected from different sources to capture a wide range of road abnormalities such as road accidents, vehicle fire, snatching, and fighting, as shown in Fig. 7. Mobile cameras and surveillance devices were used to capture photographs and videos in several Pakistani cities. This is the first openly available dataset of road anomalies from the South Asia Region. There is variability in size, form, and environmental circumstances across the many examples in the collection. For the development of intelligent transportation and surveillance systems, this dataset is of great significance. Vehicle trajectory angles were recorded in the movies, displaying the routes and directions of the vehicles^53^. Table 2 offers a summary of the RAD dataset.

**Table 2:** Key characteristics of RAD dataset Videos.

File name	Video length	Image (width × height) pixels @ frames	Lighting Situation	Class of Anomaly
RA05	8 s	640 × 520 @30	low	Road accident
RA08	12 s	1280 × 720 @25	high	Road accident
RA06	15 s	1280 × 720 @30	low	Road accident
VF05	11 s	1920 × 1080 @24	low	Car fire
VF08	17 s	1280 × 720 @24	high	Car fire
Fi09	16 s	640 × 480 @30	high	Fighting
Fi10	18 s	640 × 480 @25	low	Fighting
Sn07	22 s	1920 × 1080 @25	low	Snatching
Sn10	19 s	640 × 480 @30	high	Snatching

### UCF dataset

**Fig. 8:**
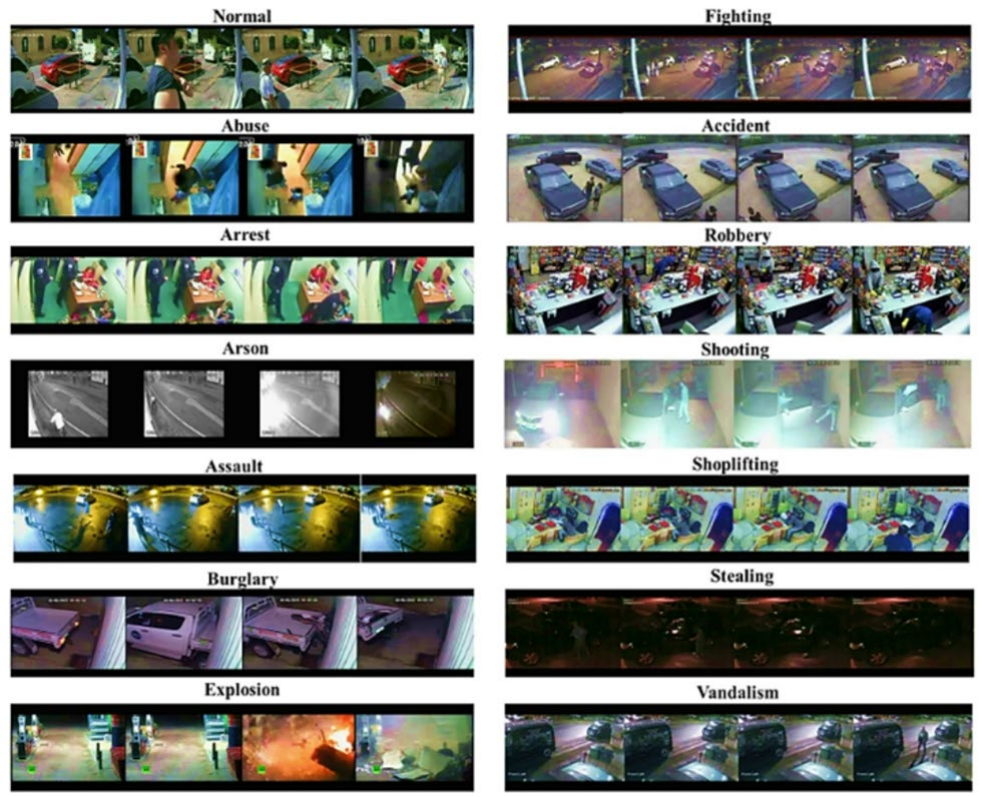
Images of the UCF dataset.

The University of Central Florida’s (UCF) dataset is used to measure how well the suggested approach works. Figure 8^54^ shows 13 distinct sorts of real-world abnormal occurrences, such as abuse, arrest, arson, assault, accidents, burglary, explosions, fighting, robbery, shooting, and vandalism. It is a realistic and extensive dataset. The fact that many of the videos are long and contain multiple scenes makes it difficult to exactly detect and identify anomalous activity.

### Model performance

Model performance is evaluated using several standard metrics, including the Receiver Operating Characteristic (ROC) curve, confusion matrix, accuracy, recall, precision, specificity, and F1-score. In anomaly detection (AD), the goal is to maximize true positives (TP) and true negatives (TN) while minimizing false positives (FP) and false negatives (FN). Here, TN refers to correctly identified normal instances, and TP indicates accurately detected anomalies. In contrast, FP arises when normal cases are misclassified as anomalies, and FN occurs when anomalies are incorrectly identified as normal. The mathematical definitions of these metrics are provided in Eqs. (3)–(7).

3 $$\:\mathrm{A}\mathrm{c}\mathrm{c}\mathrm{u}\mathrm{r}\mathrm{a}\mathrm{c}\mathrm{y}=\frac{\mathrm{T}\mathrm{P}+\mathrm{T}\mathrm{N}}{\mathrm{T}\mathrm{P}+\mathrm{F}\mathrm{P}+\mathrm{T}\mathrm{N}+\mathrm{F}\mathrm{N}}$$

4 $$\:\mathrm{P}\mathrm{r}\mathrm{e}\mathrm{c}\mathrm{i}\mathrm{s}\mathrm{i}\mathrm{o}\mathrm{n}=\frac{\mathrm{T}\mathrm{P}}{\mathrm{T}\mathrm{P}+\mathrm{F}\mathrm{P}}\:$$

5 $$\:\mathrm{S}\mathrm{e}\mathrm{n}\mathrm{s}\mathrm{i}\mathrm{t}\mathrm{v}\mathrm{i}\mathrm{t}\mathrm{y}\left(\mathrm{R}\mathrm{e}\mathrm{c}\mathrm{a}\mathrm{l}\mathrm{l}\right)=\frac{\mathrm{T}\mathrm{P}}{\mathrm{T}\mathrm{P}+\mathrm{F}\mathrm{N}}\:$$

6 $$\:\mathrm{S}\mathrm{p}\mathrm{e}\mathrm{c}\mathrm{i}\mathrm{f}\mathrm{i}\mathrm{t}\mathrm{y}=\frac{\mathrm{T}\mathrm{N}}{\mathrm{T}\mathrm{N}+\mathrm{F}\mathrm{P}}\:$$

7 $$\:\mathrm{F}1-\mathrm{S}\mathrm{c}\mathrm{o}\mathrm{r}\mathrm{e}=2\:\mathrm{x}\:\frac{\mathrm{P}\mathrm{r}\mathrm{e}\mathrm{c}\mathrm{i}\mathrm{s}\mathrm{i}\mathrm{o}\mathrm{n}\:\:\mathrm{x}\:\:\mathrm{R}\mathrm{e}\mathrm{c}\mathrm{a}\mathrm{l}\mathrm{l}}{\mathrm{P}\mathrm{r}\mathrm{e}\mathrm{c}\mathrm{i}\mathrm{s}\mathrm{i}\mathrm{o}\mathrm{n}\:+\:\mathrm{R}\mathrm{e}\mathrm{c}\mathrm{a}\mathrm{l}\mathrm{l}}\:$$

### Experimental setup

The studies in this study were carried out using a Windows-based system that has an 12 GB NVIDIA RTX 3090Ti GPU, 16 GB of RAM, and a 10th-generation Intel Core i7 CPU. CUDA 12.3.x was used to optimize GPU processing, while PyTorch 1.10.0 was used to write the Python 3.6 source code for deep learning model construction. The UCF dataset and the Road Anomaly Dataset (RAD) are used to evaluate the proposed model, and both datasets are publicly available. To keep the data uniform and easier to handle. The classification accuracy was unaffected since just 30 frames were chosen from each movie to conserve memory, evenly distributed across the video. Bounding box masks downsized to 255 × 255 pixels made up the model’s input data. The dataset was artificially expanded in size and diversity using several techniques, such as flipping, zooming, and rotating, to aid the model in learning to identify various abnormalities. Additionally, the pixel values in the frame were normalized from 0 to 1 by dividing them by 255, which scales them to a value from 0 to 1. This prepares the model for more effective training. The dataset was split into 10% testing, 20% validation, and 70% for training the model. The model was trained for 50 epochs, with a batch size of 64 and a learning rate of 1 × 10⁻⁴. Other important hyperparameters were a learning rate that decreased over time using a cosine decay schedule and a dropout rate of 0.2 to prevent overfitting.

The model had softmax as its objective function and the Adaptive Moment Estimation (Adam) algorithm as its optimizer. We adjusted the design and testing during the design phase, and after testing on real-world data with other parameter settings, we found that this parameter arrangement (3 × 3 max pooling layer, stride of 2) produced the best results for anomaly detection in surveillance videos. While they are not high-dimensional feature maps in comparison to larger feature maps, 25 × 25 feature maps strike a good balance between feature extraction and processing efficiency. By employing max pooling with a 3 × 3 filter and a stride of 2, the model can focus on the most crucial variables while boosting processing speed and performance. This effectively shrinks spatial dimensions while keeping important components.

### Data augmentation

**Fig. 9:**
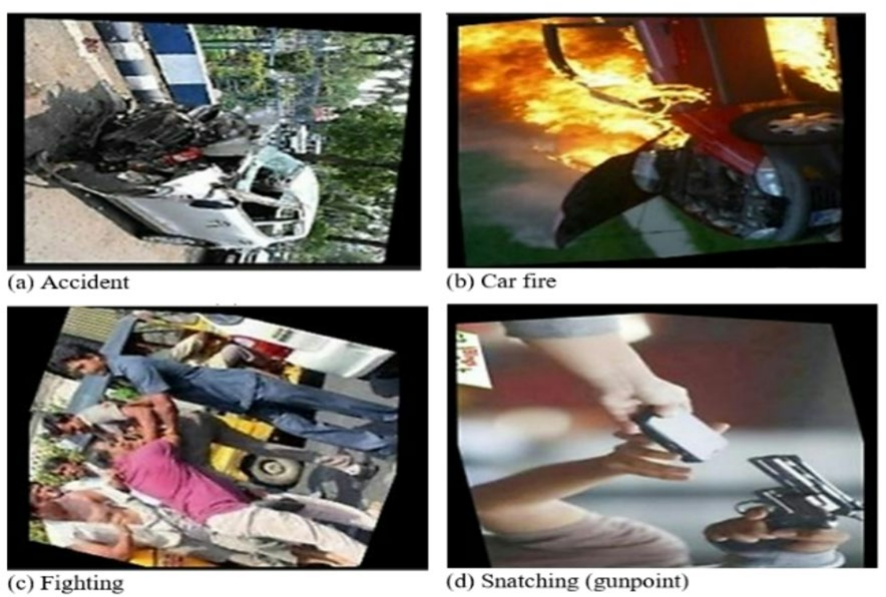
The sample images with the applied augmentation approach.

By creating copies of the original data that undergo transformations, image augmentation eliminates the requirement for further real-world data collection. Common transformations in image augmentation are translation, flipping, and rotation, as well as changing color characteristics such as saturation, contrast, and brightness, which introduce uncertainty to the data and may enable the model to learn more attributes. We augmented the data with flipping, rotation, and translation, as shown in Fig. 9. Combining these augmentation methods, we hoped to enhance the number of models to generalize across contexts.

### Results

#### UCF dataset results

The results for the four main categories (4MajCat) of the UCF dataset for the different deep learning models are presented in Table 3. The best model in terms of accuracy, precision, recall, F1-score, and specificity was InceptionV3 with 86.61%, 86.61%, 86.61%, and 86.61%, respectively. The performance evaluation of the proposed model in terms of accuracy, precision, recall, F1-score, and specificity for the four main categories (4MajCat) of the UCF dataset is given in Table 4, and it can be observed that the average accuracy, precision, recall, F1-score, and specificity were 89.90%, 89.91%, 89.90%, and 89.90%, respectively, thus validating that the proposed framework is efficient and robust in detecting abnormal events like collisions, explosions, altercations, and theft.

**Table 3:** Using four main categories on the UCF dataset, the average performance results of different deep learning models.

Model	Accuracy(%)	Precision(%)	Recall (%)	F1-Score(%)	Specificity(%)
VGG19	76.50	76.50	76.51	76.50	76.51
ResNet50	78.68	78.69	78.68	78.68	78.69
MobileNetV2	82.44	82.45	82.45	82.44	82.44
InceptionV3	86.61	86.60	86.61	86.61	86.61
DenseNet201	84.85	84.85	84.85	84.85	84.85

**Table 4:** The performance evaluation of the proposed model using four major categories on the UCF dataset.

Class	Accuracy(%)	Precision(%)	Recall (%)	F1-Score(%)		Specificity
Accident	89.91	89.91	89.90	89.90		89.90
Explosion	89.90	89.91	89.91	89.90		89.91
Fighting	89.90	89.90	89.91	89.90		89.90
Stealing	89.90	89.91	89.91	89.90		89.90
Average	89.90	89.91	89.91	89.90		89.90

**Fig. 10:**
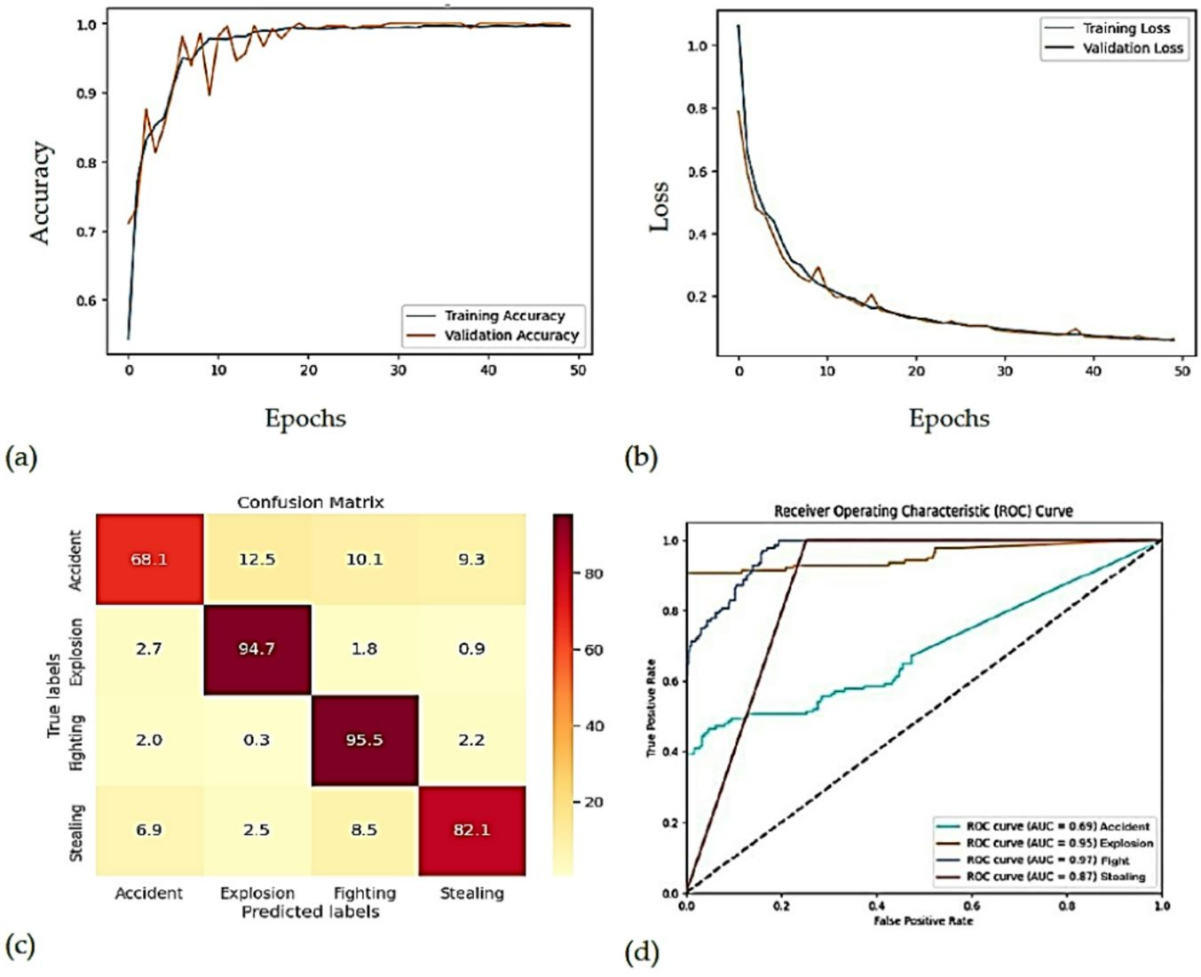
(a). Accuracy Curve (b). Loss Curve (c). Confusion Matrix (d). ROC Curve.

The proposed approach is compared with other state-of-the-art methods in Table 6, and a more comprehensive evaluation of its performance on the UCF dataset is shown in Fig. 10. Figure 10(a) shows that the validation loss is initially at 0.56 and steadily decreases to 0.26 after about 50 epochs, Fig. 10(b) shows that the validation accuracy is initially at 0.65, peaks near the 50th epoch, and increases to 0.89, subfigure 11(c) presents the confusion matrix, which demonstrates the accuracy for each class and confirms the classification performance of the model, and subfigure 11(d) shows the ROC curve, which highlights the relationship between true-positive and false-positive rates and is used to evaluate the predictive ability of the model. Together, these figures provide a comprehensive analysis of the model’s performance.

**Table 5:** Using the UCF dataset, compare the accuracy of the suggested model with earlier state-of-the-art models.

Authors, Reference	Model	Accuracy(%)
Sudhakaran et al^55^.	Convolutional LSTM	77.01
B. Cheng et al^56^.	Flow Gated Network	87.26
Y. Su et al^57^.	SPIL Convolutional Network	89.32
Z. Islam et al^58^.	SepConvLSTM-M	89.70
G.A. Martínez et al^59^.	3D CNN	75.71
A. Ansari et al^60^.	Inceptionv3 + LSTM	74.53
W. Ullah et al.^61^	CNN + LSTM	78.43
I. Muneer et al.^62^	InceptionV3 + BiLSTM	81.01
Proposed Model	CNN Encoder + Transformer Decoder	89.90

**Table 6:** Comparison of the proposed model AUC with prior studies.

Authors, Reference	Model	AUC(%)
A.O. Tur et al^63^.	K-diffusion	65.20
K. Simonyan et al^64^.	VGG-16	72.65
K. Biradar et al.^65^	DEARESt	76.65
J. X.Zhong et al^66^.	TSN-optical flow	78.09
Y.Tian et al.^67^	RTFM	84.31
Proposed Model	CNN Encoder + Transformer Decoder	90.50%

The results shown in Table 5 show that, on the UCF dataset, the suggested model achieves the highest accuracy of 89.90%. As indicated in Table 6, the suggested approach outperforms the previously researched approaches with an AUC of 90.50%. These results demonstrate the suggested approach’s superior presentation and dependability in comparison to earlier research.

#### RAD dataset results

**Fig. 11:**
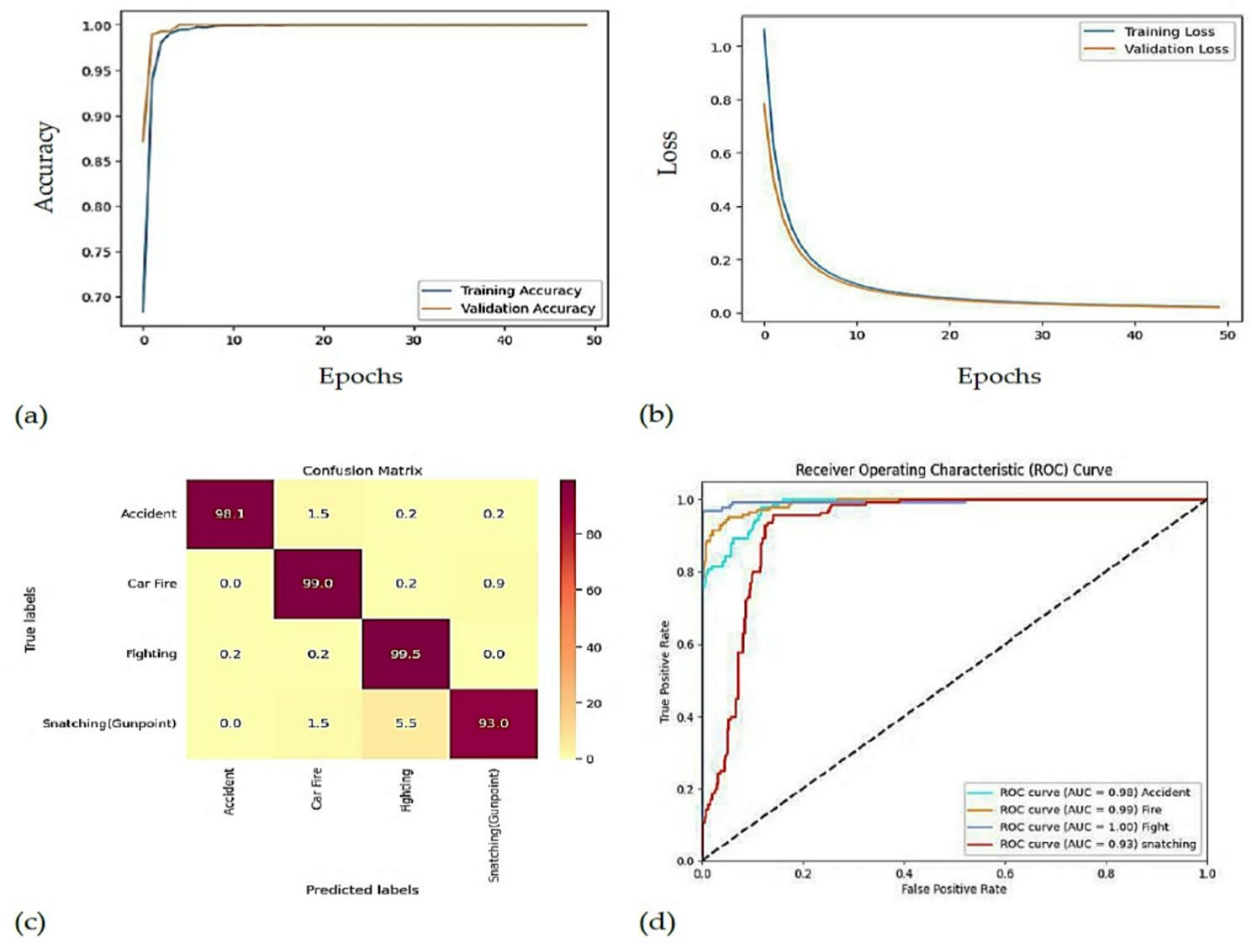
(a). Accuracy Curve (b). Loss Curve (c). Confusion Matrix (d). ROC Curve.

**Fig. 12:**
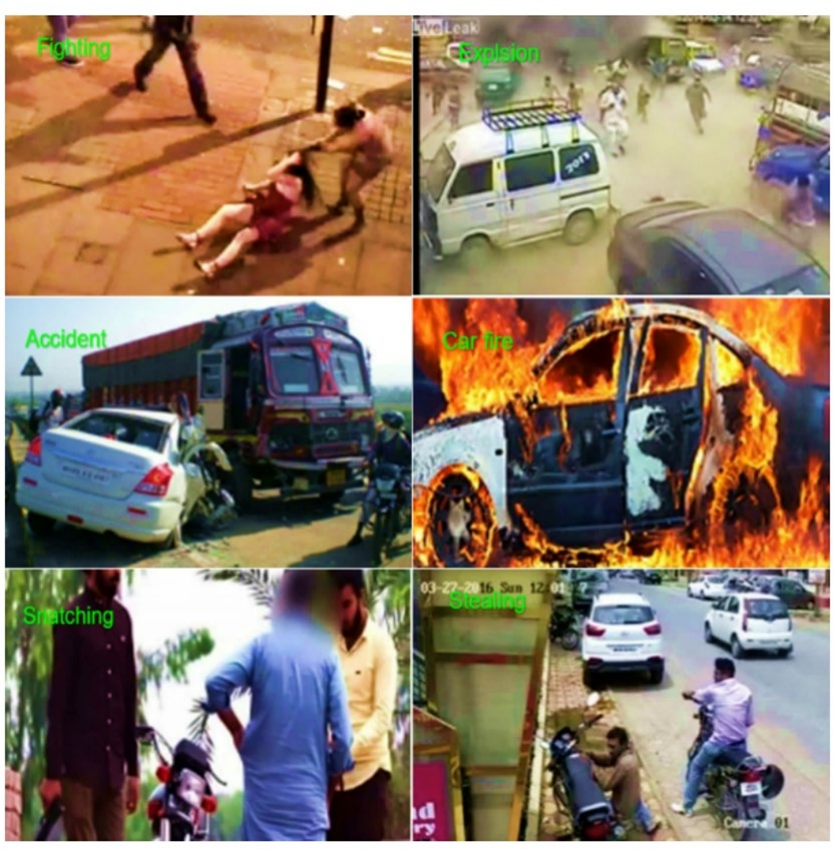
The suggested model’s test results.

Table 7 shows the performance of the deep learning models on the RAD dataset, and Table 8 shows the proposed model performance on the same dataset, which shows better results in all classes with accuracy, precision, recall, F1-score, and specificity ranging from 98.28% to 98.29% with a significant superiority over other deep learning methods. A more thorough model evaluation of the RAD dataset is presented in Fig. 11, which shows that the validation loss (Fig. 11a) begins at 0.4 and steadily decreases until it reaches the minimum at around 50 epochs, while the validation accuracy (Fig. 11b) begins at 0.85 and quickly rises to its peak around 50 epochs before gradually increasing to 0.98, the confusion matrix (Fig. 11c) shows the classification accuracy for each class and confirms the model’s robustness, and the ROC (AUC) curve (Fig. 11d) shows the trade-off between true positives and false positives that reflects the effectiveness of the model. Figure 12 presents the test results of the proposed model.

**Table 7:** The RAD dataset was used to evaluate the average performance of different deep learning models.

Models	Accuracy(%)	Precision(%)	Recall (%)	F1-Score(%)	Specificity(%)
VGG19	78.20	78.20	78.21	78.21	78.21
ResNet50	82.14	82.14	82.13	82.14	82.14
MobileNetV2	89.36	89.35	89.36	89.36	89.35
InceptionV3	90.54	90.55	90.55	90.54	90.54
DenseNet201	89.60	89.60	89.61	89.60	89.61

**Table 8:** The performance evaluation of the proposed model on the RAD dataset.

Class	Accuracy(%)	Precision(%)	Recall (%)	F1-Score(%)	Specificity(%)
Accident	98.28	98.28	98.28	98.29	98.28
Car Fire	98.29	98.29	98.29	98.28	98.29
Fighting	98.28	98.28	98.29	98.29	98.28
Snatching(gunpoint)	98.28	98.29	98.28	98.28	98.28
Average	98.28	98.28	98.28	98.29	98.28

## Conclusion and future work

An innovative approach for detecting road anomalies is presented in this study, which consists of two main stages: Anomaly Detection and Bounding-Box mask extraction. The YOLOv8 model separates objects from the background using bounding box coordinates to create focused bounding box masks to enhance object clarity and reduce background noise. The Anomaly Detection stage employs a CNN encoder to extract spatial data from these bounding box masks via convolutional and max-pooling methods, which allows the Transformer decoder to determine whether an accident has taken place within each frame. In order to quantify the advantages of the backdrop reduction, the accuracy of the framework was measured with and without this functionality. Results indicate that background removal significantly improved the accuracy and robustness of the model. This system achieved an accuracy of 98.28% on the Road Anomaly Dataset (RAD) and 89.90% on the UCF Crime dataset, which is a better result than previously reported methods. Further improvements to improve the effectiveness of real-time detection and response, future work will concentrate on improving the robustness of the model by thoroughly testing its performance in different environmental conditions, such as rain, fog, and snow, to ensure accurate anomaly detection under different weather conditions, which would lead to a prompt response in real-time, dynamic scenarios.

## Data Availability

The datasets are publicly available, Road Anomaly Dataset UCF (Crime dataset) and RAD(road anomaly dataset) https://www.kaggle.com/datasets/odins0n/ucf-crime-datasethttps://data.mendeley.com/datasets/8chk8vdn2z/1.(Accessed on 12 January 2025).
